# An Interpretable Deep Learning and Molecular Docking Framework for Repurposing Existing Drugs as Inhibitors of SARS-CoV-2 Main Protease

**DOI:** 10.3390/molecules30163409

**Published:** 2025-08-18

**Authors:** Juan Huang, Jialong Gao, Qu Chen

**Affiliations:** School of Biological and Chemical Engineering, Zhejiang University of Science and Technology, Hangzhou 310023, China

**Keywords:** deep learning, molecular docking, attention mechanism, drug repurposing, SARS-CoV-2 main protease

## Abstract

Despite the widespread use of vaccines against SARS-CoV-2, COVID-19 continues to pose global health challenges, requiring efficient drug screening and repurposing strategies. This study presents a novel hybrid framework that integrates deep learning (DL) with molecular docking to accelerate the identification of potential therapeutics. The framework comprises three crucial steps: (1) a previously developed DL model is employed to rapidly screen candidate compounds, selecting those with predicted interaction scores above a cut-off value of 0.8; (2) AutoDock Vina version 1.5.6 and LeDock version 1.0 are used to evaluate binding affinities, with a threshold of <−7.0 kcal·mol^−1^; and (3) predicted drug–protein binding sites are evaluated to determine their overlap with known active residues of the target protein. We first validated the framework using four experimentally confirmed COVID-19 drug–target pairs and then applied it to identify potential inhibitors of the SARS-CoV-2 main protease (M^Pro^). Among 29 drug candidates selected based on antiviral, anti-inflammatory, or anti-cancer properties, only Enasidenib met all three selection criteria, showing promise as an M^Pro^ inhibitor. However, further experimental and clinical studies are required to confirm its efficacy against SARS-CoV-2. This work provides an interpretable strategy for virtual screening and drug repurposing, which can be readily adapted to other DL models and docking tools.

## 1. Introduction

**Figure 1:**
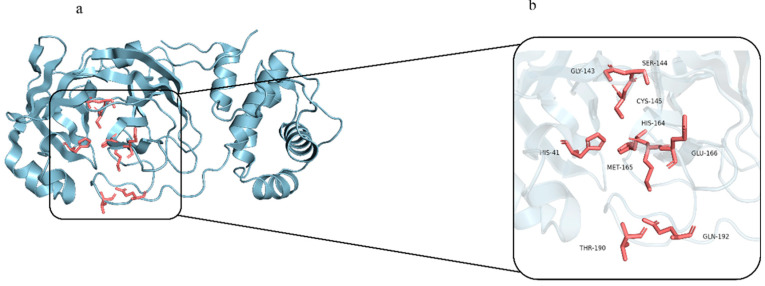
(**a**) Three-dimensional structure of SARS-CoV-2 main protease (M^Pro^); (**b**) close-up view of the M^Pro^ active site.

Persistent infection with SARS-CoV-2 remains a global health concern, following its widespread transmission during the coronavirus disease 2019 (COVID-19) pandemic [1]. Despite the extensive use of vaccines, highly mutated Omicron variants of SARS-CoV-2 continue to emerge, exhibiting immune evasion and posing risks to vulnerable populations [2]. Therefore, developing new therapeutics is still urgently needed to complement vaccination and manage ongoing COVID-19 cases. A key antiviral drug target is the main protease (M^Pro^; also known as 3C-like protease or 3CL^Pro^; PDB ID: 6LU7), which specifically recognizes and cleaves viral polyproteins pp1a and pp1ab at multiple conserved sites to release non-structural proteins essential for viral replication and maturation [3,4]. Figure 1 shows the 3D structure of M^pro^ and its active site. M^Pro^ features a highly conserved substrate-binding pocket across coronaviruses [4]. This conservation ensures that both the substrate recognition sites and the 3D structure of M^Pro^ remain nearly unchanged among different coronaviruses, making it an ideal target for the development of broad-spectrum antiviral drugs [5]. Moreover, because human cells lack homologous proteases, the risk of off-target toxicity is minimized [5]. Nirmatrelvir, a component of the oral antiviral Paxlovid (Nirmatrelvir + Ritonavir), was approved by the U.S. Food and Drug Administration (FDA) for emergency use in treating COVID-19. It functions by directly binding to the M^Pro^ active site, thereby inhibiting the main protease’s activity and suppressing viral replication within host cells [6].

Several other FDA-approved drugs target different sites within the SARS-CoV-2 virus, including Remdesivir, Baricitinib, and Dexamethasone. Remdesivir targets the RNA-dependent RNA polymerase (RdRp) of SARS-CoV-2 (PDB ID: 6M71), a key enzyme essential for viral RNA replication [7]. Inside host cells, the prodrug Remdesivir is metabolized into its active triphosphate form, which functions as a nucleotide analog. This active metabolite is then incorporated by RdRp into the nascent viral RNA chain, leading to premature termination of RNA synthesis and effectively inhibiting viral replication [8]. Baricitinib, a Janus kinase (JAK2) inhibitor (PDB ID: 6WTO), targets the JAK-STAT signaling pathway to modulate cytokine-driven immune responses [9,10,11]. Suppressing this pathway helps to reduce the hyperinflammation and immune overactivation seen in severe COVID-19 cases, particularly protecting lung function [12]. Dexamethasone is a corticosteroid that binds to intracellular glucocorticoid receptors (PDB ID: 1M2Z) to regulate gene expression and suppress the production of pro-inflammatory cytokines [13]. This mechanism helps mitigate the severity of cytokine storms and acute lung inflammation associated with severe COVID-19 [14].

Despite the availability of approved drugs, the continual emergence of resistant variants and limited efficacy in certain populations reveal a pressing need for novel therapeutics that can robustly inhibit target proteins. Unfortunately, traditional drug development is a resource-intensive endeavor, typically requiring over a decade and approximately USD 3 billion to bring a single new drug to market [15]. Although computational techniques have accelerated early-stage discovery, they do not fundamentally reduce the time needed for preclinical studies and clinical trials [16]. Alternatively, drug repurposing, which involves identifying new uses for existing drugs with established safety profiles, offers a more time-efficient route to drug development [17]. Computational methods enable rapid screening of repurposing candidates, making this approach particularly valuable for urgent health challenges such as ongoing infectious COVID-19. Ambrosio et al. reported a successful case of drug repurposing for an M^Pro^ inhibitor using in silico approaches. Through docking analysis, they identified betrixaban as one of the most promising candidates from a pool of approximately 8000 FDA-approved drugs, a finding that was subsequently supported by in vitro experiments [18]. Although repurposed drugs still undergo preclinical and clinical evaluation, the process is generally faster and more cost-effective than developing novel compounds from scratch [19].

In recent years, a wide range of machine learning (ML) models, from classical ML to deep learning (DL), have emerged to improve drug–target interaction (DTI) prediction [20,21,22]. These models are typically trained on large-scale datasets, extract features from sequences and structures of proteins/ligands, and perform binary classification or affinity prediction tasks. Classical ML methods, such as *k*-nearest neighbor (*k*-NN), random forest (RF), and support vector machine (SVM), often rely on manual feature engineering and face scalability issues with large datasets [22]. In contrast, deep learning approaches, such as recurrent neural network (RNN) [23], convolutional neural network (CNN) [24], and graph neural network (GNN) [25], can automatically learn high-level representations and capture complex nonlinear relationships between drugs and targets. The introduction of attention mechanisms has further advanced DTI prediction by assigning varying weights to different regions of the input, enabling models to focus on the most relevant features [26,27]. Attention also enhances interpretability by highlighting which atoms or residues contribute most to binding, thereby improving both model transparency and biological insight [28].

While ML-based models are data-driven and effective at capturing complex patterns, they often lack explicit physical interpretations of atomic-level binding events. In contrast, docking-based approaches use the 3D structures of drugs and proteins to simulate binding conformations based on the lock-and-key model and estimate interaction energies using physics-based scoring functions [29]. These methods can reveal specific interaction types in DTIs, including van der Waals (vdW) interactions, electrostatic interactions, and hydrogen bond (H-bond) interactions. As such, docking serves as a valuable complement to ML models in computational drug screening. Commonly used docking tools include AutoDock [30], AutoDock Vina [31,32], LeDock [33], Glide [34,35], and FlexX [36], among others [37], each employing distinct algorithms and scoring strategies to predict binding poses and affinities. Indeed, a growing number of studies have successfully integrated data-driven and physics-based approaches to enhance the accuracy and interpretability of DTI prediction [38,39,40].

In this work, we propose a new framework that combines a DL model with established molecular docking tools to enhance DTI predictions. Our previously developed model, AMMVF-DTI (Drug–Target Interactions Based on Attention Mechanism and Multi-View Fusion), integrates multi-view interaction modeling and attention mechanisms to capture both fine-grained (node-level) and global (graph-level) features via interaction transformers (ITMs) and neural tensor networks (NTNs) [41]. Despite its strong performance, the model has some limitations: first, it relies solely on 1D sequence information for both drugs and proteins, neglecting 3D structural features essential for accurate DTI modeling [42]; second, it lacks atomic-level interpretations of DTIs, hence requiring further visualization of the attention mechanism. To address these issues, we incorporate docking-based approaches, which offer both physics-based insights and explicit 3D structural representations of molecular interactions. This hybrid strategy aims to improve predictive performance and provide mechanistic insights for drug discovery and repurposing.

This paper is organized as follows. Section 2.1 and Section 2.2 present the validation and application of our proposed hybrid framework, respectively. Specifically, Section 2.1 validates the combined DL and docking approach by screening FDA-approved drugs (Remdesivir, Nirmatrelvir, Baricitinib, and Dexamethasone) against their respective target proteins. Section 2.2 discusses drug repurposing results targeting M^Pro^, with subsequent validation through molecular dynamics (MD) simulations. Section 3 outlines our hybrid framework that integrates DL and molecular docking for DTI prediction. Section 3.1 introduces the DL model and compares its performance against several existing models, while Section 3.2 describes the two docking programs employed in this study (AutoDock Vina and LeDock) and the way to prepare input files. Finally, the key findings and implications of this study are summarized in Section 4.

## 2. Results and Discussion

### 2.1. Framework Validation with Known Drug–Target Pairs

To validate our proposed DL–docking framework for screening potential drugs, we selected four COVID-19-related drugs (Nirmatrelvir, Remdesivir in its triphosphate form, Baricitinib, and Dexamethasone) with their target proteins, as discussed in the Introduction. Additionally, we included three negative samples: Chloroquine, Amoxicillin, and Cephradine. These drugs have been clinically tested and evaluated through experimental or computational studies but show no meaningful interaction with M^Pro^. Chloroquine, initially developed as an antimalarial agent, has shown no significant clinical benefit in treating COVID-19, despite early interest in its potential antiviral effects [43]. Using the fluorescence resonance energy transfer (FRET)-based enzymatic assay, Ma and Wang explicitly stated that chloroquine is not an inhibitor of the SARS-CoV-2 main protease [44]. Amoxicillin and Cephradine, both antibiotics, have not been shown to improve clinical outcomes in COVID-19 patients [45]. Ghahremanpour et al. identified 42 candidates as inhibitors of M^pro^ from approximately 2000 approved drugs by a consensus virtual screening protocol, and neither Amoxicillin nor Cephradine was in the list of the selected potential candidates, suggesting weak or no interaction between M^pro^ and these drugs [46].

For seven known drug–target pairs, we first used our DL model to predict their potential interactions and then employed AutoDock Vina version 1.5.6 and LeDock version 1.0 to calculate the binding affinities of their best-predicted poses. The predicted scores and binding affinities are presented in Table 1. Our DL model demonstrated greater reliability in screening potential drugs than the two docking models. Specifically, for Nirmatrelvir, Remdesivir triphosphate (Remdesivir-TP), Baricitinib, and Dexamethasone, the DL model accurately predicted interactions with their respective experimentally validated target proteins, yielding predicted scores above 0.8. Note that, in a binary classification DL model for DTI, as opposed to a regression model, a higher prediction score typically reflects a higher probability of interaction, not necessarily a stronger binding affinity [47,48]. Conversely, for Chloroquine, Amoxicillin, and Cephradine, the DL model correctly predicted limited interactions with the irrelevant protein M^Pro^. However, the binding affinities calculated by the docking models were highly diverse and less useful for drug screening. For example, AutoDock Vina predicted relatively strong affinities (−7.4 kcal·mol^−1^) for Amoxicillin and Cephradine with M^Pro^, despite their lack of reported interactions. It also predicted a comparatively weaker affinity (−6.6 kcal·mol^−1^) for Nirmatrelvir with M^Pro^, which is a known target. LeDock similarly predicted comparable affinities for Dexamethasone and Cephradine with their tested proteins, even though only Dexamethasone is known to interact with its target. Note that we define binding free energies lower than −7.0 kcal·mol^−1^ as indicative of strong binding affinity in this study. This empirical threshold is commonly regarded as “strong binding” in the field of molecular docking and has been widely adopted in the literature [49,50,51].

The explanation for this discrepancy is straightforward: for complex and flexible proteins, although docking programs can predict drug–target interactions, the predicted binding may not occur at the protein’s active sites and hence may not inhibit its catalytic activity. To investigate this, we further analyzed which residues of M^Pro^ are involved in binding Chloroquine, Amoxicillin, and Cephradine as predicted by AutoDock Vina and LeDock. Table 2 presents the binding site predictions from the two docking programs. For the antimalarial Chloroquine, the results predicted from the DL model and the docking programs are in excellent agreement; the binding affinities predicted from the two docking programs were relatively low (greater than −6 kcal·mol^−1^). For the antibiotics Amoxicillin and Cephradine, AutoDock Vina predicted strong binding affinities (less than −7.0 kcal·mol^−1^), but none of the identified binding residues were located at or near the well-known active sites of M^Pro^ (e.g., His41, Cys145, His164, and Thr190) [52,53]. Conversely, while LeDock did predict some residues (e.g., Gly143, Ser144, Cys145, and Glu166) at or near these active sites, the overall binding affinities were comparatively low (~−5.8 kcal·mol^−1^). These combined results suggest that Chloroquine, Amoxicillin, and Cephradine are unlikely to be effective M^Pro^ inhibitors, which is consistent with the predictions from our DL model.

**Figure 2:**
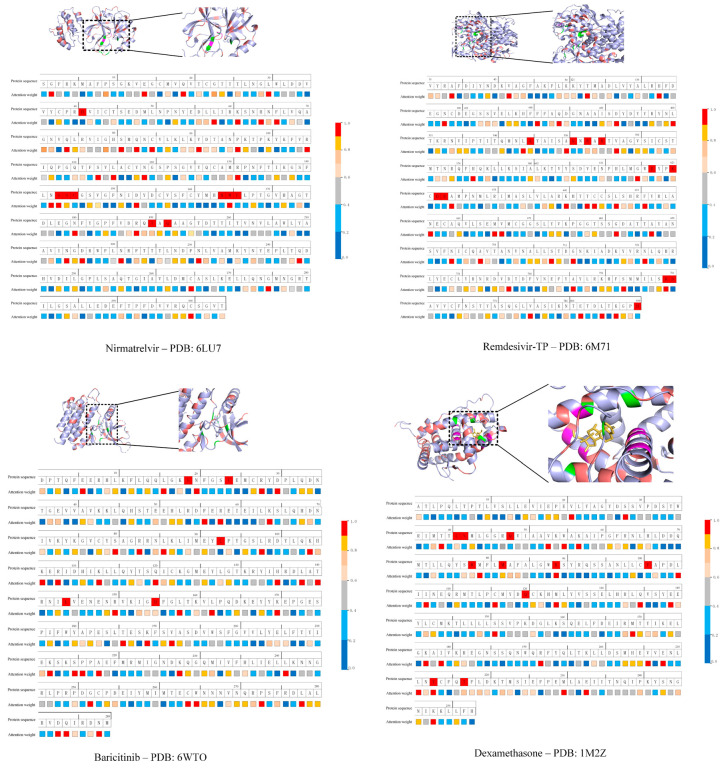
Visualization of the attention weights learned by our DL model for four drug–protein pairs: Nirmatrelvir and PDB:6LU7, Remdesivir triphosphate (Remdesivir TP) and PDB:6M71, Baricitinib with PDB:6WTO, and Dexamethasone with PDB:1M2Z. In the “Attention weight” row, colors indicate the attention assigned to individual residues (red: high weight; blue: low weight; scale shown on the right). Residues marked in red in the “Protein sequence” row correspond to experimentally validated binding sites. Additional details on high-attention residues are provided in Table S1 of the Supplementary Information.

Moreover, we compared the predictions of our framework for the four well-known drug–target pairs with experimental data to further validate its performance. While the DL model successfully identified interactions for all four pairs (see Table 3), it was still necessary to further evaluate whether the attention mechanism assigned significant weights to the actual key residues of these proteins. To this end, we visualized the attention weights learned by our DL model, shown in Figure 2, with detailed data provided in Table S1 of the Supplementary Information. In our analysis, residues with attention weights over 80%, marked in red or bright yellow on the color scale, were considered highly attended. For comparison, experimentally verified binding residues are marked in red in the “Protein residue” row of Figure 2. For M^Pro^ (PDB ID: 6LU7), the DL model identified Asn142, Gly143, Ser144, Met165, Ala191, and Ala193, the residues known to be at or adjacent to key active sites [52]. For RdRp (PDB ID: 6M71), the critical residues Leu544, Lys545, Ala550, Ala625, Ser759, and Asp760 were identified [53]. Similarly, for JAK2 (PDB ID: 6WTO), residues Asn859 and Ile982 were identified [54], and for the glucocorticoid receptor (PDB ID: 1M2Z), residues Met601, Arg611, Tyr735, Met565, Met604, and Cys643 were identified [13]. It should be noted that, in this study, we consider the identification of residues adjacent to known binding sites as a successful prediction, given the structural complexity and flexibility of the proteins investigated. Small deviations in residue selection may still reflect meaningful biological relevance, particularly when the binding interface is dynamic or when neighboring residues contribute indirectly to ligand binding. These findings demonstrate that, for all four drug–target pairs, the high-attention residues predicted by our DL model overlap with experimentally validated binding sites. This suggests that our model has basically learned to recognize biologically relevant features at the residue level. However, we acknowledge that the attention mechanism we employed, when limited to 1D sequence or graph features and aimed solely at predicting overall binding probability, tends to produce broad attention distributions. Potential solutions include introducing sparsity constraints to encourage more focused attention and incorporating known binding site annotations as supervision to guide the model toward learning biologically meaningful attention patterns centered on actual binding residues.

Although our DL model seems to have learned key structural patterns underlying DTIs, the large number of residues assigned high attention weights limits its ability to precisely identify binding sites, especially in the absence of experimental data. Furthermore, attention weights reflect only 1D sequence-level information, whereas functional binding regions are typically defined by the protein’s secondary and tertiary structures. In contrast, molecular docking programs provide atomistic, 3D information on binding site residues and predicted affinities. As mentioned earlier, binding affinities below −7.0 kcal·mol^−1^ are typically considered strong, while values above this threshold may suggest weak or nonspecific interactions [49,50,51]. As shown in Table 4, AutoDock Vina and LeDock both predicted good affinities for four well-known DTIs (i.e., Nirmatrelvir–M^Pro^, Remdesivir triphosphate–RdRp, Baricitinib–JAK2, and Dexamethasone–glucocorticoid receptor), except for AutoDock’s slightly weaker prediction for the first pair and LeDock’s weaker prediction for the last pair (>−7.0 kcal·mol^−1^).

**Figure 3:**
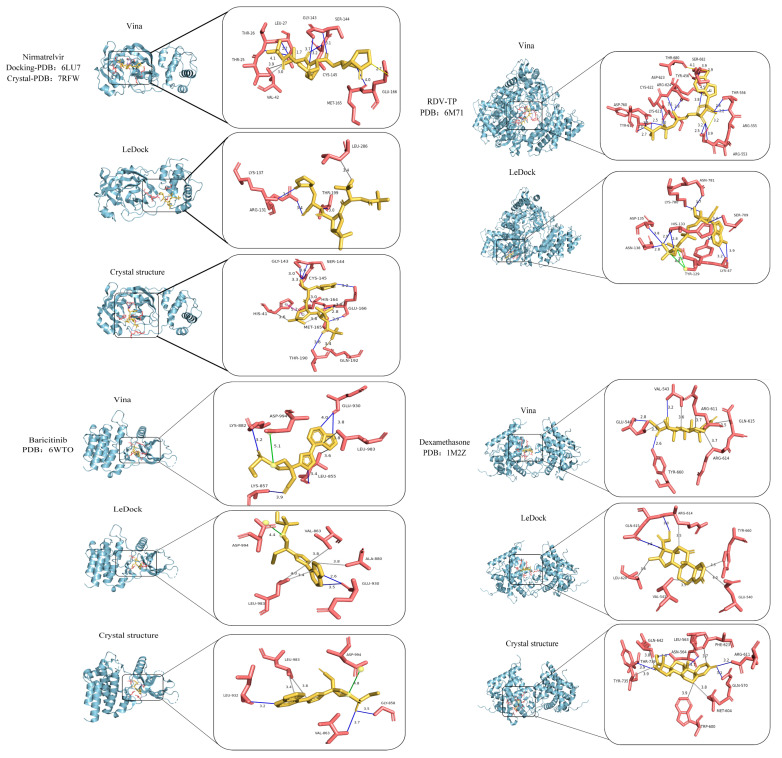
Best binding poses for four drug–protein pairs (i.e., Nirmatrelvir–M^Pro^, Remdesivir triphosphate–RdRp, Baricitinib–JAK2, and Dexamethasone–glucocorticoid receptor) using LeDock and AutoDock Vina. For each pair, the overall binding conformation is shown on the left, while a close-up view of the binding site is shown on the right. In the close-up images, protein residues are colored red, drug molecules are yellow, hydrogen bonds are shown as blue lines, and hydrophobic interactions are shown as grey lines.

To evaluate whether the docking programs captured experimentally validated binding sites, we compared their predicted residues with known crystal structure data (shown in Figure 3 and Table 3). In Table 3, hydrogen-bonded residues are shown in black; salt-bridge-only residues in red; and hydrophobic-only residues in blue. For Remdesivir triphosphate–RdRp, where direct crystal data are unavailable, we referenced key residues supported by experimental evidence [53]. A glance at Figure 3 clearly reveals that AutoDock Vina generally captured the binding pose of Nirmatrelvir–M^Pro^, while both docking programs correctly predicted the binding conformation of Baricitinib–JAK2. More specifically, in Table 3, for the pair Nirmatrelvir–M^Pro^, AutoDock Vina successfully identified key M^Pro^ residues (Val42, Gly143, Ser144, Cys145, and Glu166) at or adjacent to the binding sites for Nirmatrelvir, while LeDock did not. For the pair Remdesivir triphosphate–RdRp, LeDock correctly predicted several key residues (Arg553, Arg555, Thr556, Tyr619, Lys621, Cys622, Asp623, Arg624, and Asp760), whereas AutoDock Vina did not. Both programs captured important binding residues Leu983 and Asp994 for the pair Baricitinib–JAK2, while neither of them fully captured the correct binding sites for the pair Dexamethasone–glucocorticoid receptor, with only AutoDock Vina partially identifying Arg611. These results show a key challenge: no single docking tool consistently outperforms others across all protein targets [55,56]. Since the proteins tested here represent diverse protein families ranging from viral enzymes (e.g., RdRp) to human signaling proteins (e.g., JAK2), the accuracy of docking predictions can vary depending on the protein type and docking algorithm. Therefore, combining multiple docking tools offers a more robust strategy for improving predictive reliability and generating biologically meaningful insights.

### 2.2. Predictive Application to COVID-19 Main Protease

Based on the results from four positive and three negative drug–protein pairs, we found that DL models are effective for rapidly identifying potential drug candidates for a given target protein, and molecular docking programs can further examine the binding sites and affinities between the protein and selected drugs. Accordingly, we propose three steps to use our hybrid framework that integrates our DL model with molecular docking tools for virtual drug repurposing.

Step 1: DL Filter. Apply the DL model to rapidly screen candidate drugs and retain those with high predicted scores (with a binary default cut-off value of 0.5) based on learned features. This step filters out compounds with little or no predicted interaction with the target protein.

Step 2: Docking Affinity Filter. Use AutoDock Vina and LeDock to calculate binding affinities for the top-scoring poses and select drug–protein pairs with predicted affinities below −7.0 kcal·mol^−1^. This step eliminates compounds with weak predicted affinity for the target.

Step 3: Binding Pose Validation. Compare the predicted binding residues of the protein to experimentally validate active sites to assess whether the candidate drug is likely to inhibit protein function. This step helps identify candidate drugs that not only bind strongly but also bind to the correct active site of the target protein.

It is important to note that our deep learning model performs a binary classification task, where typically a prediction score above 0.5 indicates a positive interaction, while a score below 0.5 suggests no interaction. However, in this work, we used an empirical cut-off value of 0.8 based on the model’s performance across multiple benchmark datasets, where it consistently demonstrated stable and reliable predictions at or above this level. Despite the adjustment, using this threshold as the initial filter presents a key limitation of the hybrid framework when applied to large-scale drug screening. Specifically, a substantial number of compounds may receive scores above 0.8 for a given target, making subsequent docking computations labor-intensive and time-consuming.

In contrast, our hybrid model is better suited for evaluating a relatively small set of drug candidates, where the proposed three-step process can efficiently find the most promising compounds. Consequently, we selected 29 drug candidates for potential repurposing, most of which are FDA-approved, with the exceptions of Favipiravir, Daclatasvir, Carmofur, and Masitinib, which are approved in Japan or/and Europe. Unlike the three negative control compounds discussed earlier that have been clinically verified ineffective against COVID-19, these drugs exhibit potential therapeutic activity against SARS-CoV-2 but lack direct experimental or clinical evidence confirming their interactions with SARS-CoV-2-related proteins. For consistency and comparison, we focused on evaluating their interactions with SARS-CoV-2 M^Pro^, although several of these drugs may also act on other targets such as RdRp, JAK2, or intracellular glucocorticoid receptors. The selection of these compounds was based on their known pharmacological categories as antiviral, anti-inflammatory, and anti-cancer, as previous studies have suggested that compounds in these categories might demonstrate inhibitory effects on SARS-CoV-2 [57,58,59,60,61]. Among the 16 antiviral drugs, Favipiravir, Oseltamivir, Zanamivir, and Peramivir are used to treat influenza; Lamivudine, Efavirenz, Lopinavir, and Ritonavir are used against human immunodeficiency virus (HIV); Sofosbuvir, Velpatasvir, Voxilaprevir, Daclatasvir, Glecaprevir, Pibrentasvir, and Ribavirin against hepatitis C virus (HCV); and Acyclovir against herpesvirus. Among the seven anti-inflammatory drugs, Tofacitinib, Sirolimus, Imiquimod, and Ibuprofen are classified as anti-inflammatory or immune-modulating agents, while Diclofenac, Naproxen, and Indomethacin are classified as nonsteroidal anti-inflammatory drugs (NSAIDs). Among the six anti-cancer drugs, Enasidenib, Nilotinib, Dasatinib, and Imatinib are primarily approved for the treatment of various forms of leukemia; Carmofur for the treatment of colon cancer; and Masitinib for pancreatic cancer.

We used our proposed three-step framework to evaluate the 29 repurposed drug candidates, with the results summarized in Table 4. In Step 1, our DL model identified 14 drugs with high predicted interaction scores (≥ 0.8), namely, Zanamivir, Peramivir, Lamivudine, Sofosbuvir, Voxilaprevir, Glecaprevir, Pibrentasvir, Tofacitinib, Enasidenib, Nilotinib, Dasatinib, Imatinib, Carmofur, and Masitinib. In Step 2, AutoDock Vina and LeDock identified 8 of these 14 compounds as having binding affinities below −7.0 kcal·mol^−1^ in at least one docking program. These drugs include Voxilaprevir, Glecaprevir, Pibrentasvir, Enasidenib, Nilotinib, Dasatinib, Imatinib, and Masitinib. The results suggest that three antiviral and five anti-cancer drugs show promising binding potential to M^Pro^, but few anti-inflammatory drugs were successfully selected after Steps 1 and 2. This may be due to the fact that anti-inflammatory drugs are more likely to act on immune-related targets such as JAK1/2 [9].

**Figure 4:**
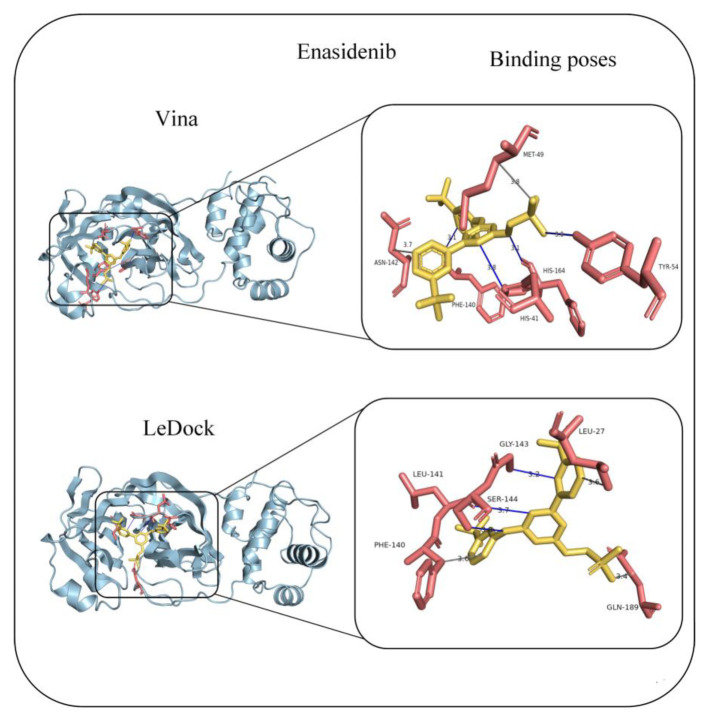
Predicted binding poses and binding residues between Enasidenib and M^pro^ using AutoDock Vina and LeDock.

Step 3 is crucial for evaluating whether the selected drug candidates bind to the functionally relevant active sites of M^Pro^ by virtual docking programs. According to crystallographic studies (PDB IDs 6LU7 and 7RFW), residues His41, Gly143, Ser144, Cys145, His164, Met165, Glu166, Thr190, and Gln192 form the core of the active site [4,6,52]. Binding to these residues is considered essential for effective inhibition of the main protease’s catalytic activity. We show the binding site predictions for three selected antiviral drugs (Voxilaprevir, Glecaprevir, and Pibrentasvir) with M^Pro^ in Figure S1 and those for four selected anticancer drugs (Nilotinib, Dasatinib, Imatinib, and Masitinib) in Figure S2 of the Supplementary Information. The results indicate that none of these compounds bind to the active site or adjacent residues in either AutoDock Vina or LeDock predictions. This means that despite favorable binding affinities, these drugs are unlikely to inhibit M^Pro^ function due to their binding positioning outside the protein’s catalytic site. The only compound that passed all three steps of our framework is Enasidenib, an FDA-approved isocitrate dehydrogenase 2 (IDH2) inhibitor used in the treatment of IDH2-mutant acute myeloid leukemia. As shown in Figure 4, AutoDock Vina predicts that Enasidenib interacts with His41, Asn142, and His164—residues located at or near the M^Pro^ active site. Similarly, LeDock identifies interactions with Asn142 and Glu166, both at or adjacent to the catalytic core. Our finding is in excellent agreement with another in silico report by Anwaar et al. [62], who also identified Enasidenib as a potential M^Pro^ inhibitor in their drug repurposing study for COVID-19. Nevertheless, further experimental validation and clinical trials are essential to confirm its antiviral efficacy and assess its safety profile in treating SARS-CoV-2 infections.

Finally, several other limitations of this study should be discussed. First, the performance of our hybrid framework depends on the predictive reliability of the DL model, which serves as the primary screening filter. Given its “black-box” nature, the DL model may exclude potentially effective drug candidates due to limitations in robustness and generalizability. Enhancing model training on broader, high-quality datasets would be essential to reduce false negatives. Second, molecular docking tools typically neglect protein flexibility and solvent effects, and incorporating molecular dynamics (MD) simulations and binding free energy calculations could substantially improve predictive accuracy [63,64,65,66]. We present additional results from MD simulations of Enasidenib-Mpro complex in Note S1 of the Supplementary Information. Despite these limitations, the proposed hybrid framework offers a promising in silico strategy for drug repurposing and, when complemented with experimental validation, holds potential for accelerating therapeutic discovery.

## 3. Materials and Methods

**Figure 5:**
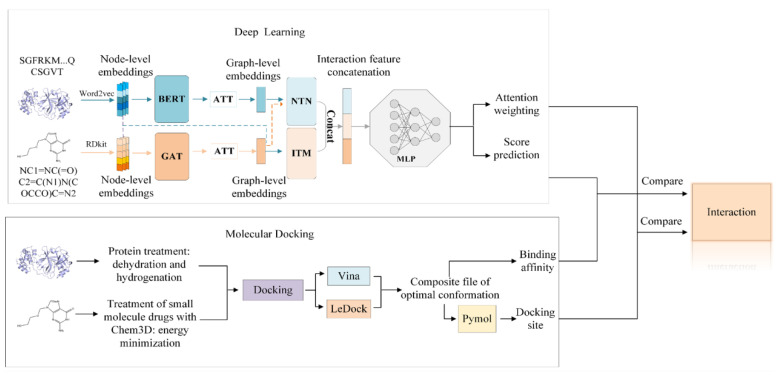
Scheme of our interpretable DL and molecular docking framework for DTI predictions.

The overall workflow of our interpretable DL and molecular docking framework is illustrated in Figure 5. The framework first employs our DL model to rapidly screen and select candidate ligands with high predicted affinity, followed by molecular docking to evaluate their binding modes and interaction strengths. By integrating these two approaches, the framework aims to reduce the computational cost of docking while improving the predictive accuracy and interpretability of the DL model.

### 3.1. Deep Learning Model

The DL model architecture is composed of three stages: feature extraction, interaction modeling, and prediction. In the feature extraction stage, raw drug and protein data—namely, SMILES (Simplified Molecular Input Line Entry System) strings [67] and amino acid sequences—are transformed into node-level embeddings, as they cannot be directly processed by the model. Specifically, the cheminformatics toolkit RDKit [68] is used to convert SMILES strings into molecular graphs and extract atom-level features. For protein sequences, a pretrained Word2Vec model [69] is used to encode residues into numerical vectors that capture local sequence patterns. These embeddings form the input features for subsequent stages of the DL model.

In the interaction modeling stage, the node-level embeddings are first processed through two distinct modules: a Graph Attention Network (GAT) [70] for drugs and a Bidirectional Encoder Representations from Transformers (BERT) [71] module for proteins. These modules extract contextualized, high-dimensional features that capture the local structural and sequential information of drugs and proteins, respectively. Next, a multi-head attention mechanism (ATT) [26] is subsequently applied to aggregate the node-level outputs into graph-level representations that capture higher-order structural information. To enhance the model’s ability to capture diverse types of drug–target interactions, both node-level and graph-level features are fed into two specialized interaction modules: interaction transformers (ITMs) and neural tensor networks (NTNs). The ITM is responsible for learning pairwise interactions by modeling complex dependencies between atoms in the drug and protein residues. It applies cross-attention between the two sets of embeddings, enabling the model to capture fine-grained local interactions that are essential for binding specificity. In contrast, the NTN captures global and nonlinear relationships by modeling bilinear interactions between the overall graph-level representations of drugs and proteins. The NTN effectively learns potential interaction patterns that might not be obvious from local attention alone. Together, the ITM and NTN complement each other: ITM emphasizes localized structural and chemical interactions, while NTN focuses on global compatibility between molecules.

In the prediction stage, the learned interaction features (both local atomic-level and global structural-level information from the drug and protein) are combined and input into a multilayer perceptron (MLP) [72] for final DTI prediction. The MLP consists of multiple fully connected layers, each followed by nonlinear activation functions (e.g., ReLU), dropout for regularization, and batch normalization to stabilize training. Through these layers, the model learns complex, nonlinear mappings between interaction patterns and the likelihood of drug–target binding. The final output layer uses a sigmoid activation function to generate a probability score between 0 and 1, representing the predicted likelihood of interaction between the drug and protein. A threshold (typically 0.5 in binary classification tasks) is then applied to determine whether the interaction is predicted to be positive or negative. For more detailed architectural and methodological information, readers are referred to our original work [41].

We evaluated the performance of our DL model via five-fold cross-validation on three benchmark datasets: Human, *C. elegans*, and DrugBank. The Human and *C. elegans* datasets are relatively small, while DrugBank is a large-scale dataset [73]. Table S2 of the Supplementary Information summarizes the information in the datasets used for training the DL model. Table S3 of the Supplementary Information outlines the key hyperparameters used to train our deep learning model for DTI prediction. The protein and atom embeddings were set to dimensions of 100 and 34, respectively, while the hidden layer size was 64. The model included three layers of GAT, each with three attention heads, and used eight heads for the multi-head self-attention mechanism. Structural features were captured using a radius of 2- and 3-g tokenization. A dropout rate of 0.1 was applied to prevent overfitting. The model was trained with a batch size of 32, a learning rate of 1 × 10^−3^, and a regularization coefficient of 1 × 10^−4^. Additionally, the number of major potential associations (*K*) was set to 16, and training was carried out over 40 epochs.

The average area under curve (AUC), precision, and recall values for our model and for several benchmark methods are reported in Table 5, Table 6 and Table 7. On the Human and *C. elegans* datasets, our model outperformed traditional ML baselines such as KNN, RF, L2, and SVM, as well as several DL baselines such as MDL-CPI [74], GNN [75], graph convolutional network (GCN), GraphDTA [76], DrugVQA(VQA-seq) [47], and TransformerCPI [27]. On the DrugBank dataset, our model outperformed several established models, including RWR (ML-based) [77], DrugE-Rank (ML-based) [78], GNN-CPI (DL-based) [28], and DeepCPI (DL-based) [79], and showed performance comparable to that of GraphDTA (DL-based) [76]. These results show the relative strength of our model in DTI prediction. We acknowledge that the performance metrics (AUC, precision, and recall) of our model remain modest when trained on the larger DrugBank dataset. First, in contrast to smaller and more homogeneous human and *C. elegans* datasets, DrugBank is a large-scale and highly heterogeneous dataset, encompassing a wide variety of compound and protein types. Such a large public databases often contain noise and inconsistencies, which can affect the model’s ability to learn reliable patterns. Second, our current model primarily relies on 1D representations for drugs and amino acid sequences for proteins. Although this approach works well on smaller datasets, it may be insufficient for capturing the more diverse and complex DTI patterns present in DrugBank.

However, it is important to note that the objective of this work is not to outperform all existing models but rather to demonstrate that our model is qualified for DTI prediction and can serve as an important component within the hybrid DL and docking framework illustrated in Figure 5. Moreover, using our DL model is generally convenient and efficient. For model training, smaller datasets such as human and *C. elegans* take approximately 5 min per training epoch on a standard GPU server. For larger datasets like DrugBank, a full training cycle takes roughly 30 min. Once the model is trained, scoring new drug–target pairs is instantaneous, making it feasible to rapidly evaluate thousands of combinations.

### 3.2. Molecular Docking Programs

Wang et al. conducted a comprehensive evaluation comparing ten docking programs across diverse protein–ligand complexes [55]. They found that the academic program LeDock ranked among the top two for scoring power following the commercial program GOLD. Moreover, AutoDock Vina demonstrated the best overall scoring performance among all the tested programs. Therefore, we utilized the academic programs LeDock and AutoDock Vina in this work. First, AutoDock [30] and AutoDock Vina [31,32] are two widely used computational tools for molecular docking, both developed by the Olson Laboratory at The Scripps Research Institute. While AutoDock employs a genetic algorithm (GA) and a Lamarckian Genetic Algorithm (LGA) to search for optimal binding conformations of the ligand, AutoDock Vina integrates a stochastic global search with a local optimization method based on the Broyden–Fletcher–Goldfarb–Shanno (BFGS) algorithm for more efficient sampling of the conformational space [31,80]. Moreover, AutoDock Vina features a re-designed scoring function that incorporates a broader range of physicochemical interactions, enhancing its ability to estimate binding affinities. Compared to AutoDock, AutoDock Vina significantly improves docking speed and is particularly effective in high-throughput virtual screening campaigns.

Second, LeDock [33] employs a hybrid optimization algorithm that integrates an LGA with a local search strategy, enabling the program to explore a wide conformational space of ligands within a reasonable computational time. For each sampled conformation, a scoring function is used to estimate the binding free energy, thereby predicting the most favorable binding mode between a ligand and its target protein. The scoring function combines empirical physicochemical parameters to evaluate key intermolecular interactions, such as van der Waals forces and electrostatic interactions. Its robust performance and speed make it particularly well suited for high-throughput virtual screening.

The docking simulation procedure was conducted as follows. Initially, we selected and downloaded the protein crystal structure from the Protein Data Bank (PDB), choosing PDB ID: 6LU7 for the SARS-CoV-2 main protease (M^pro^), for example. This structure was selected for two main reasons: first, it provides high-resolution data (2.16 Å), allowing precise characterization of the key active site residues (His41–Cys145) and the surrounding binding pocket; second, it is one of the most widely used M^pro^ structures in virtual screening, molecular dynamics, and drug design studies [63,65,66]. For protein preparation, AutoDockTools (ADT) version 1.5.6 was used to process the PDB file by removing all bound ligands, cofactors, and water molecules, followed by the addition of polar hydrogens to define the protein as the receptor. For ligand preparation, we downloaded the molecular structures from PubChem in .mol format, performed energy minimization in Chem3D [81], converted them to .pdb format using PyMOL version 2.6 [82], and then used ADT to add hydrogens, automatically detect rotatable bonds, and generate .pdbqt files. During grid generation, we set a grid box encompassing the entire protein, which slightly reduces docking speed but ensures that no potential binding site is overlooked.

## 4. Conclusions

In this study, we present a hybrid framework that integrates DL and molecular docking to repurpose existing drugs as inhibitors of M^pro^. Our framework consists of a three-step virtual screening pipeline. In the first step, our previously developed DL model was employed to rapidly filter drug candidates predicted to interact with the target protein, selecting those with interaction scores above 0.8. We confirmed that the DL model captures biologically relevant features by demonstrating overlaps between the high-attention residues it identified and experimentally verified binding sites. In the second step, two widely used docking programs, AutoDock Vina and LeDock, were used to calculate binding affinities, and drugs with predicted affinities stronger than −0.7 kcal·mol^−1^ were retained. In the third step, predicted binding sites using two docking tools were evaluated to determine whether the drug candidates could bind to the known active sites of the target protein. This framework is particularly effective for identifying inhibitors of proteins with experimentally resolved 3D structures and their active sites.

To validate our framework, we first applied it to four clinically relevant drug–target pairs associated with COVID-19: Nirmatrelvir–MPro, Remdesivir–RdRp, Baricitinib–JAK2, and Dexamethasone–glucocorticoid receptor. The inclusion of multiple and diverse proteins helps demonstrate the robustness of this approach. We then applied the framework to a set of 29 approved drugs, selected based on their reported antiviral, anti-inflammatory, or anti-cancer properties that may be associated with COVID-19 therapeutic strategies. Through this in silico analysis, an anti-cancer agent Enasidenib was finally identified as a promising M^Pro^ inhibitor, with predicted binding at or near the protein’s active site. Overall, our hybrid framework provides a generalizable and interpretable strategy for virtual drug screening and repurposing, particularly in response to emerging infectious diseases.

## Figures and Tables

**Table 1:** Predicted scores and binding affinities of seven drug–protein pairs from our DL model and two docking models, respectively. Note that Remdesivir-TP represents its triphosphate form that actually functions.

DrugID	PDBID	DL Score	Docking (kcal·mol^−1^)	
			AutoDock Vina	LeDock
Nirmatrelvir	6LU7	0.994	−6.6	−7.26
Remdesivir-TP	6M71	0.991	−8.0	−9.26
Baricitinib	6WTO	0.828	−8.8	−7.52
Dexamethasone	1M2Z	0.972	−8.0	−5.25
Chloroquine	6LU7	0.065	−5.6	−4.92
Amoxicillin	6LU7	0.001	−7.4	−5.89
Cephradine	6LU7	0.007	−7.4	−5.81

**Table 2:** Predicted binding sites between the main protease (PDB ID: 6LU7) and three drugs (Chloroquine, Amoxicillin, and Cephradine) using AutoDock Vina and LeDock. Note that no results on salt bridges or π-stacking interactions were provided by LeDock.

DrugID	H-Bonding Interactions		Hydrophobic Interactions		Salt Bridge	π-Stacking
	Vina	LeDock	Vina	LeDock	Vina	Vina
Chloroquine	n/a	Gln110 Asn151	Phe8 Ile106 Gln110 Asn151 Phe294	Phe8 Asn151 Phe294	Asp153	n/a
Amoxicillin	Gln110 Thr111 Asp295	Thr26 Leu141 Gly143 Ser144 Cys145 Glu166	Val104 Asn151	Thr25 Glu166	n/a	Phe294
Cephradine	Gln110 Thr111 Asn151 Asp295	Asn142 Gly143 Ser144 Cys145 His172	Val104 Asn151	Glu166	n/a	Phe294

**Table 3:** Predicted binding residues for four drug–target protein pairs using AutoDock Vina and LeDock. Residues shown in black indicate hydrogen bond interactions between proteins and their respective drugs. Residues highlighted in red represent exclusively salt-bridge interactions, while those in blue indicate exclusively hydrophobic interactions.

DrugID	PDBID	AutoDock Vina	LeDock	Crystal Structure
Nirmatrelvir	6LU7	Thr24, Thr25, Thr26, Leu27, Val42, Gly143, Ser144, Cys145, Glu166	Arg131, Lys137, Thr199, Leu286	His41, Gly143, Ser144, Cys145, His164, Met165, Glu166, Thr190, Gln192
Remdesivir-TP	6M71	Lys47, Tyr129, His133, Asp135, Asn138, Ser709, Lys780, Asn781	Tyr456, Arg553, Arg555, Thr556, Tyr619, Lys621, Cys622, Asp623, Arg624, Thr680, Ser682, Asp760	Lys545, Lys551, Arg553, Arg555, Asp618, Lys621, Asp623, Arg624, Asp760, Asp761 His810 (predicted)
Baricitinib	6WTO	Leu855, Lys857, Lys882, Glu930, Leu983, Asp994	Val863, Ala880, Glu930, Leu983, Asp994	Gly858, Val863, Leu932, Leu983, Asp994
Dexamethasone	1M2Z	Glu540, Val543, Arg611, Arg614, Gln615, Leu620, Tyr660	Glu540, Val543, Arg614, Gln615, Leu620, Tyr660	Leu563, Asn564, Gln570, Trp600, Met604, Arg611, Phe623, Gln642, Tyr735, Thr739

**Table 4:** Binding scores predicted by our DL model and binding affinities predicted by two docking programs (AutoDock Vina and LeDock) for the interactions between 29 repurposed drugs and SARS-CoV-2 M^Pro^. Scores over 0.8 are highlighted in red, and affinities below −7 kcal·mol^−1^ are highlighted in blue.

DrugID	Drug Action	DL Score	Docking (kcal·mol^−1^)	
			AutoDock Vina	LeDock
Favipiravir	antiviral	0.003	−5.1	−4.5
Oseltamivir	antiviral	0.115	−5.3	−4.8
Zanamivir	antiviral	0.836	−6.7	−6.8
Peramivir	antiviral	0.882	−6.1	−5.3
Lamivudine	antiviral	0.991	−5.7	−5.5
Efavirenz	antiviral	0.028	−7.2	−4.5
Lopinavir	antiviral	0.558	−7.2	−6.9
Ritonavir	antiviral	0.451	−7.2	−7.9
Sofosbuvir	antiviral	0.922	−4.8	−6.6
Velpatasvir	antiviral	0.781	−8.9	−7.7
Voxilaprevir	antiviral	0.924	−8.8	−6.8
Daclatasvir	antiviral	0.223	−8.3	−8.1
Glecaprevir	antiviral	0.875	−8.6	−6.5
Pibrentasvir	antiviral	0.839	−7.7	−7.4
Ribavirin	antiviral	0.003	−6.4	−5.7
Acyclovir	antiviral	0.572	−5.3	−6.1
Tofacitinib	anti-inflammatory	0.968	−6.1	−5.8
Sirolimus	anti-inflammatory	0.388	−8.9	−6.8
Imiquimod	anti-inflammatory	0.005	−6.8	−4.6
Ibuprofen	anti-inflammatory	0.141	−6.0	−3.6
Diclofenac	anti-inflammatory	0.001	−6.4	−4.7
Naproxen	anti-inflammatory	0.335	−6.5	−4.0
Indomethacin	anti-inflammatory	0.005	−6.9	−5.0
Enasidenib	anti-cancer	0.878	−9.2	−7.6
Carmofur	anti-cancer	0.956	−5.6	−5.2
Masitinib	anti-cancer	0.990	−8.2	−7.42
Nilotinib	anti-cancer	0.979	−9.3	−7.4
Dasatinib	anti-cancer	0.997	−7.1	−7.4
Imatinib	anti-cancer	0.991	−8.3	−7.4

**Table 5:** Comparison of AUC, precision, and recall values for our model and other models on the Human dataset.

Model	AUC	Precision	Recall
KNN	0.860	0.927	0.798
RF	0.940	0.897	0.861
L2	0.911	0.913	0.867
SVM	0.910	0.966	0.969
MDL-CPI	0.959	0.924	0.905
GNN	0.970	0.923	0.918
GCN	0.956	0.862	0.928
GraphDTA	0.960	0.882	0.912
DrugVQA(VQA-seq)	0.964	0.897	0.948
TransformerCPI	0.973	0.916	0.925
**This work**	**0.989**	**0.958**	**0.987**

**Table 6:** Comparison of AUC, precision, and recall values for our model and other models on the *C*. *elegans* dataset.

Model	AUC	Precision	Recall
KNN	0.858	0.801	0.827
RF	0.902	0.821	0.844
L2	0.892	0.890	0.877
SVM	0.894	0.785	0.818
MDL-CPI	0.975	0.943	0.929
GNN	0.978	0.938	0.929
GCN	0.975	0.921	0.927
GraphDTA	0.974	0.927	0.912
TransformerCPI	0.988	0.952	0.953
**This work**	**0.989**	**0.981**	**0.963**

**Table 7:** Comparison of AUC, precision, and recall values for our model and other models on the DrugBank dataset.

Model	AUC	Precision	Recall
RWR	0.760	0.705	0.651
DrugE-Rank	0.759	0.707	0.629
GNN	0.802	0.737	0.716
DeepCPI	0.700	0.700	0.556
GraphDTA	0.821	0.751	0.769
**This work**	**0.815**	**0.775**	**0.760**

## Data Availability

The original contributions presented in this study are included in the article. Further inquiries can be directed to the corresponding author.

## Supplementary Materials

The following supporting information can be downloaded at: https://www.mdpi.com/article/10.3390/molecules30163409/s1, Figure S1: Predicted binding residues between M^pro^ and three antivirial drugs; Figure S2: Predicted binding residues between M^pro^ and four anti-cancer drugs; Table S1: List of highly weighted residues identified by our DL model; Table S2: Summary of the three datasets used in this study; Table S3: Hyperparameter setting for our deep learning model; Note S1: Molecular dynamics simulations of Enasidenib-M^pro^ complex [83,84,85,86,87]. The Python code for our deep learning model and for visualizing residues with varying attention weights is available as open source and can be downloaded from the following website: https://github.com/frankchenqu/hybrid (accessed on 1 July 2025).
