# Supplementary material for: An Interpretable Deep Learning and Molecular Docking Framework for Repurposing Existing Drugs as Inhibitors of SARS-CoV-2 Main Protease
Source: Molecules. 2025 Aug 18;30(16):3409. doi: 10.3390/molecules30163409 (PMC12388385; doi:10.3390/molecules30163409)
Supplement: Supplementary file 1 [file molecules-30-03409-s001.zip › molecules-3763501-supplementary.pdf]

Table of Contents

Table S1. List of highly weighted residues identified by our DL model.

Figure S1. Predicted binding residues between M^{pro} and three antivirial drugs.

Figure S2. Predicted binding residues between M^{pro} and four anti-cancer drugs.

Table S2. Summary of the three datasets used in this study.

Table S3. Hyperparameter setting for our deep learning model.

Note S1. Molecular dynamics simulations of Enasidenib-M^{pro} complex

Table S1. List of highly weighted residues identified by our deep learning (DL) model for four drug–target pairs. Residues highlighted in red have attention weights over 90%, while those in yellow have weights over 80%. Double-underlined residues indicate exact matches to known binding residues from crystal structures; single-underlined residues indicate those adjacent to these binding residues (e.g. if residue 142 is a binding residue, residues 141 and 143 are considered adjacent).

DrugID	PDBID	Highly weighted residues
Nirmatrelvir	6LU7	Gly2, Glu14, Gln19, Leu30, Leu58, Lys61, His64, Phe66, Gln74, Ser81, Gln83, Cys85, Val91, Pro96, Arg105, Ser113, Leu115, Asn119, Arg131, Asn142, Gly143, Asp153, Met165, Asn180, Arg188, Asn214, Asp216, Asn221, Asn231, Gln244, Cys265, Gly275, Cys300<br>Thr24, Cys37, Asn53, Leu75, Leu87, Lys97, Lys100, Gln107, Tyr118, Pro122, Ile136, Gly138, Ser144, Ser147, Asn151, Val157, Pro168, His172, Pro184, Ala191, Ala193, Gly215, Thr224, Asn228, Asp229, Asp248, Leu250, Gln256, Leu262, Ser284, Arg298, Gln299
Remdesivir triphosphate	6M71	Ala34, Val42, Leu119, Thr123, His133, Asn138, Phe441, Gln444, Asp452, Leu460, Leu544, Ala550, Val557, Gly616, Ala625, Lys641, Tyr644, Cys645, Leu648, Phe652, Asn657, Met666, Val667, Gly683, Ala688, Asn695, Ala699, Ser709, Gly712, Asn722, Asp738, Ala747, Ser754, Asp760, Val776<br>Asn39, Lys438, Ile450, Asn459, Lys532, Val535, Gln541, Lys545, Ala558, Ser561, Thr565, Arg569, Ala580, Ser607, Ala639, Cys646, Gln661, Tyr674, Val675, Thr680, Ala706, Leu707, Thr710, Tyr719, Tyr732, Asn734, Asp736, Asn743, Ays751, His752, Ser759, Phe766, Tyr770, Ser778, Leu786, Asn790, Asn791
Baricitinib	6WTO	Glu845, Leu855, Asn859, Arg867, Asp869, Phe895, Arg897, Lys903, Ser919, Arg922, Leu925, Leu937, Lys943, Lys945, Arg947, Lys952, Gln959, His974, Leu977, Thr979, Arg989, Leu997, Leu1001, Lys1009, Thr1027, Lys1030, Val1033, Lys1053, Ser1056, Pro1057, Ala1059, Lys1069, Leu1082, Gly1093, Cys1105, Trp1106, Pro1114, Leu1119, Asp1124, Gln1125, Asn1129<br>Thr842, Leu849, Phe851, Gln853, Cys866, Gln872, Asp873, Ala880, His886, His891, Leu892, His907, Lys912, Lys914, Cys917, Lys926, Glu930, Gly935, His950, Tyr956, Cys961, Glu965, Gly968, Ile982, Asn988, Asp1004, Tyr1007, Ile1018, Tyr1021, Pro1023, Glu1028, Ser1032, Ala1034, Gln1070, Gln1072, Val1075, Pro1095, Asn1107, Asn1108, Asn1111, Gln1112, Ala1120, Leu1121
Dexamethasone	1M2Z	Val543, Ser550, Thr561, Val575, Trp577, Lys579, Met601, Arg611, Leu627, Ile629, Cys638, Met646, Gln657, Glu661, Ser673, Met691, Lys699, Ile701, Arg714, Val728, Glu730, Leu733, Tyr735, Met752, Glu755, Lys770<br>Leu532, Glu537, Glu540, Pro541, Met565, Gly583, Arg585, Leu587, Met604, Leu621, Arg633, Cys643, Leu647, Cys665, Thr692, Glu696, Lys703, Met725, His726, Glu727, Ser765, Asn768, Leu773

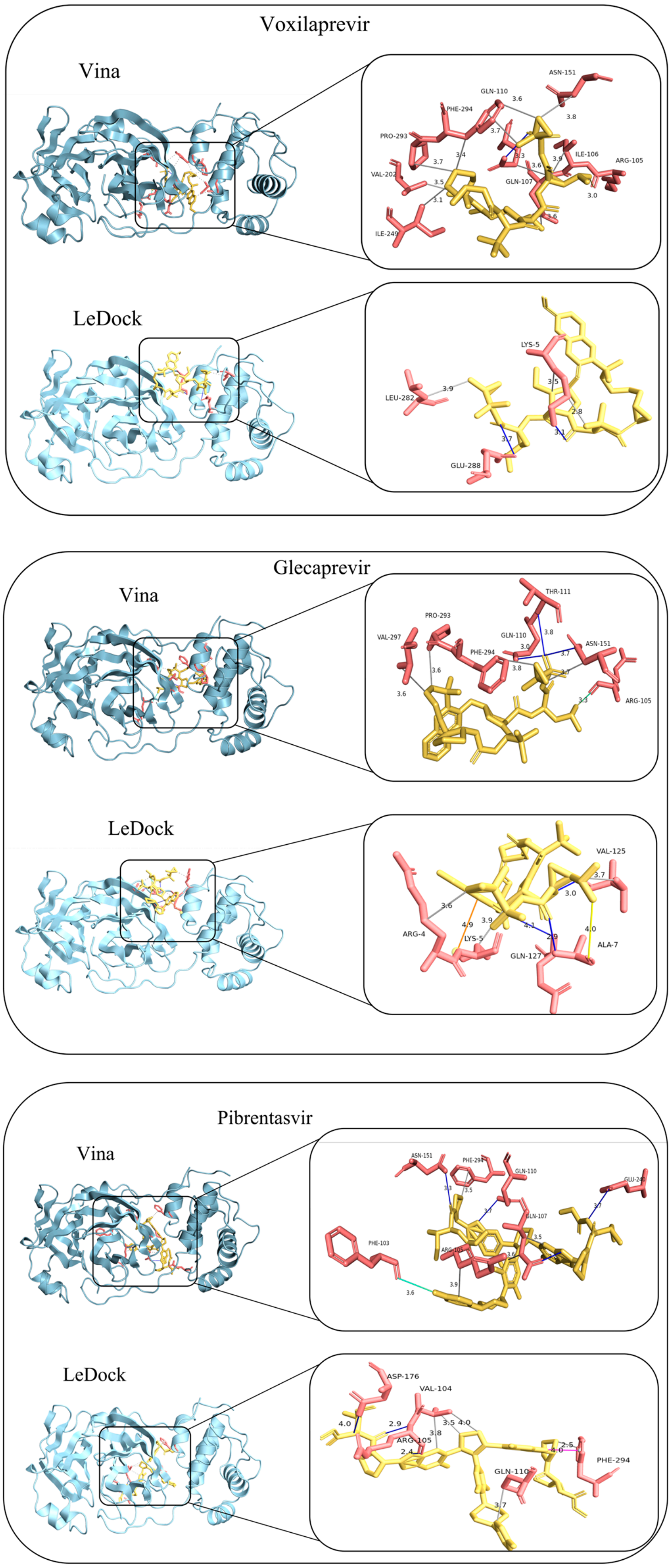

Figure S1: Predicted binding residues between SARS-CoV-2 M^{pro} and three antivirial drugs (Voxilaprevir, Glecaprevir, and Pibrentasvir) using AutoDock Vina and LeDock.

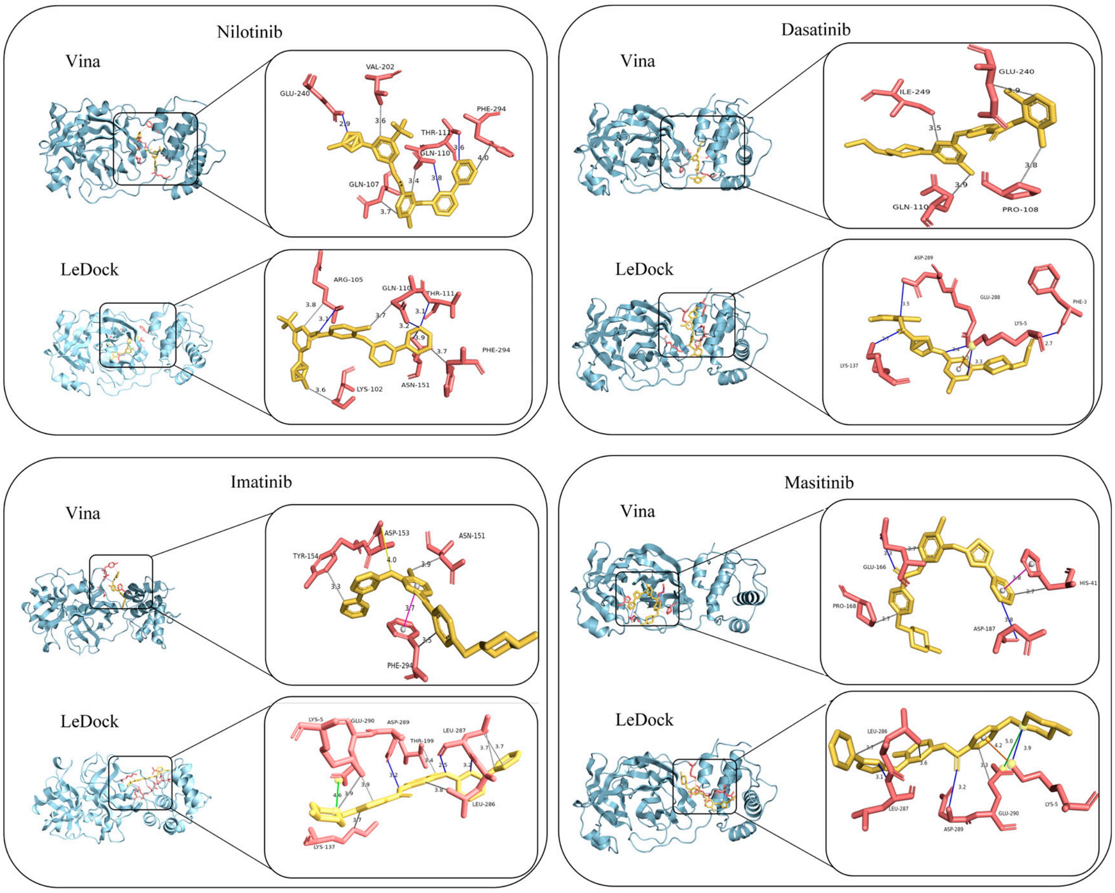

Figure S2: Predicted binding residues between SARS-CoV-2 M^{pro} and four anti-cancer drugs (Nilotinib, Dasatinib, Imatinib, and Masitinib) using AutoDock Vina and LeDock.

Table S2. Summary of the three datasets used in this study, namely Human, *C.elegans*, and DrugBank.

	Human	*C.elegans*	DrugBank
Number of drugs	1052	1434	6645
Number of target proteins	852	2504	4254
Number of total samples	6728	7786	35022
Number of positive interactions	3364	3893	17511

Table S3. Hyperparameter setting for our deep learning model.

Hyperparameter	Value
The dimensions of the protein	100
The dimensions of the atom	34
The dimensions of the hidden layer	64
The number of GAT layers	3
The number of multi-head self-attention	8
The number of heads of the GAT	3
The radius	2
The *n*-gram	3
Dropout	0.1
The batch size	32
Learning rate	1×10^{-3}
Regularization coefficient	1×10^{-4}
The number of major potential associations *K*	16
Epoch	40

Note S1. The docking programs used in this study, AutoDock Vina and LeDock, do not account for flexible protein structures or explicit water models. As a result, it is important to complement docking with molecular dynamics (MD) simulations in explicit solvent to obtain a more realistic description of protein–drug interactions. We exacted the coordinate file of the Enasidenib-M^{pro} complex from AutoDock Vina and used it as the starting configuration for further MD simulation. The M^{pro} protein was modeled with the CHARMM36 force field[1], while parameters for Enasidenib were generated using SwissParam 2023[2]. The system was solvated with explicit modified TIP3P water molecules, and counter ions were added to neutralize the total charge. Periodic boundary condition (PBC) was used in all directions. The Lennard-Jones (LJ) parameters for cross interactions and the electrostatic interactions were calculated using the Lorentz-Berthelot combining rules[3] and the particle mesh Ewald (PME) method[4], respectively. Van der Waals and electrostatic interactions were truncated at a cutoff distance of 1.2 nm. All MD simulations were performed using Gromacs 4.6.5[5]. After energy minimization, the system was under equilibration with a 0.5-ns NVT simulation followed by a 0.5-ns NPT simulation. The equilibrated structure was then subjected to a 100-ns production run. To evaluate the stability of the complex, we computed the root mean square deviation (RMSD) of Enasidenib relative to the M^{pro} binding pocket. As shown in Figure below, Enasidenib (highlighted in green) remained bound within the binding pocket of M^{pro} (highlighted in purple). The RMSD stabilized within ~0.1 nm after 30 ns, suggesting that the protein–drug complex was relatively stable over the course of the simulation.

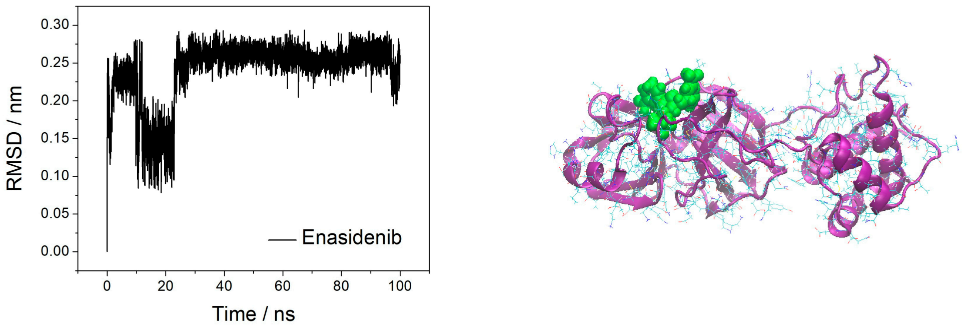

Figure S3. (left panel) RMSD of Enasidenib with respect to the binding pocket of M^{pro}; (right panel) conformation of the Enasidenib-M^{pro} complex, with water molecules not displayed for clarity.

References

1. Huang, J.; Rauscher, S.; Nawrocki, G.; Ran, T.; Feig, M.; de Groot, B.L.; Grubmuller, H.; MacKerell Jr., A.D. CHARMM36m: an improved force field for folded and intrinsically disordered Proteins. Nat. Methods 2017, 14, 71–73.

2. Bugnon, M.; Goullieux, M.; Röhrig, U.F.; Perez, M.A.S.; Daina, A.; Michielin, O.; Zoete, V. SwissParam 2023: A Modern Web-Based Tool for Efficient Small Molecule Parametrization. J. Chem. Inf. Model. 2023, 63, 6469−6475.

3. Allen, M.P.; Tildesley, D.J. Computer Simulation of Liquids. first ed., Clarendon, Oxford, 1989.

4. Darden, T.; York, D.; Pedersen, L. Particle mesh Ewald: an $N \cdot \log(N)$ method for Ewald sums in large systems. J. Chem. Phys. 1993, 98, 10089−10092.
5. van der Spoel, D.; Lindahl, E.; Hess, B.; Groenhof, G.; Mark, A.E.; Berendsen, H.J.C. GROMACS: fast, flexible, and free, J. Comput. Chem. 2005, 26, 1701−1718.
